# A station-based concept for teaching the neurological examination: A prospective quasi-experimental study

**DOI:** 10.3205/zma001076

**Published:** 2016-11-15

**Authors:** Jochen Brich, Michael Rijntjes

**Affiliations:** 1 Department of Neurology and Neuroscience, Medical Center – University of Freiburg, Freiburg i. Br., Germany

**Keywords:** neurological examination, small group teaching, self-assessment

## Abstract

**Background:** The neurological examination is considered to be complex and contributes to the phenomenon of “neurophobia”. It is traditionally taught in small groups by residents (“traditional concept”), making the learning success partially dependent on the resident’s level of clinical training, didactic education and personal motivation. Aim of this study was to examine the effects of a newly developed concept (“station concept”) for teaching the neurological examination on achieving an improved and more equal transfer of knowledge and practical skills.

**Methods: **A prospective quasi-experimental design was used to compare the traditional concept with the newly developed station concept, in which the teaching content was divided in eight subdivisions (stations) with one resident being assigned to one station. The primary endpoints of the study were the differences in students’ self-assessments of learning success in the different subdomains of the neurological examination, and secondary analyses focused on evaluation results of students and residents.

**Results:** 144 students and 28 residents participated in the traditional concept (summer semester 2012) and 151 students and 28 residents in the station concept (winter semester 2012/13). In the station-concept students’ self-assessment significantly improved in the domains “Motor System”, “Coordination” and “Mental Status” compared to the traditional concept. Students’ evaluation showed significant improvement in five out of eight points. Fifty percent of residents rated the new approach superior to the traditional approach, ten percent as inferior.

**Conclusion:** The station concept improved students’ self-assessed learning success as well as evaluation results while simultaneously achieving high acceptance in residents.

## Zusammenfassung

**Hintergrund: **Die neurologische Untersuchung gilt als komplex und trägt zum Phänomen der „Neurophobie“ bei. Sie wird traditionell in Kleingruppen durch Assistenzärzte unterrichtet („traditionelles Konzept“), wodurch der Lernerfolg teilweise vom klinischen Ausbildungsstand, der didaktischen Ausbildung und der persönlichen Motivation des Assistenzarztes abhängt. Ziel der Studie war es, die Effekte eines neu entwickelten Konzeptes („Stationen-Konzept“) für die Lehre der neurologischen Untersuchung hinsichtlich eines verbesserten und gleichmäßigeren Transfers von Wissen und praktischen Fertigkeiten zu untersuchen.

**Methoden: **Ein prospektives, quasi-experimentelles Design wurde angewandt, um das traditionelle Konzept mit dem Stationen-Konzept zu vergleichen. Dabei wurde der Lehrinhalt in acht Teilbereiche (Stationen) unterteilt wurde, wobei ein Assistenzarzt zu jeder Station zugeteilt wurde. Die primären Endpunkte der Studie waren die Unterschiede in der Selbsteinschätzung der Studierenden hinsichtlich des Lernerfolges in unterschiedlichen Bereichen der neurologischen Untersuchung. Sekundäre Analysen bezogen sich auf die Ergebnisse der Evaluationen der Studierenden und der Assistenzärzte.

**Ergebnisse: **144 Studierende und 28 Assistenzärzte nahmen am traditionellen Konzept teil (Sommersemester 2012) und 151 Studierende und 28 Assistenzärzte am Stationen-Konzept (Wintersemester 2012/13). Beim Stationen-Konzept verbesserten sich die Selbsteinschätzungen der Studierenden im Vergleich zum traditionellen Konzept signifikant in den Bereichen „Motorisches System“, „Koordination“ und „Mentaler Status“. Die Evaluationen der Studierenden ergaben signifikante Verbesserungen in fünf von acht Punkten. Fünfzig Prozent der Assistenzärzte bewerteten den Stationen-Ansatz besser als den traditionellen Ansatz, zehn Prozent als schlechter.

**Schlussfolgerung: **Das Stationen-Konzept verbesserte den von den Studierenden selbst eingeschätzten Lernerfolg wie auch die Evaluationsergebnisse bei gleichzeitig hoher Akzeptanz bei den Assistenzärzten.

## Introduction

The neurological examination (NE) is considered to be difficult and complex and contributes to the internationally recognized phenomenon of “neurophobia” [1], [2], [3], [4], [5], [6]. Nevertheless the correct performance and interpretation of the results of the NE is still of great significance, since history and the NE are the basis for the topical diagnosis in neurology, which remains important for clinical decision making also in the era of cross sectional imaging.

The content [7], [8] and different approaches of teaching the NE (hypothesis-driven or screening NE [9]) have been addressed by educational studies, but apart from anecdotal essays (for example [10]) to our knowledge no systematic studies about educational strategies for teaching the NE to undergraduate students exist.

Although lacking good evidence [11], most medical schools traditionally use the small group interactive learning approach with residents as teachers. One characteristic of this teaching approach is the close relationship between the resident as a teacher and the group of students, which creates a mostly well appreciated tight and individual mentoring. But even after completion of a didactical training – which is not obligatory in German universities - this close relationship still can cause a strong dependency on the resident’s individual qualifications concerning the level of postgraduate education and professional experience. This may result in an uncontrollable and heterogeneous learning outcome for the students. Under these circumstances a fair and objective assessment of the NE for example with an Objective Structured Clinical Examination (OSCE) [12] could be hampered, which was an important obstacle for implementation of an OSCE in our institution.

To overcome these limitations we developed a station concept for teaching the NE. The aim of this prospective quasi-experimental study was to examine the effects of the station concept on students’ self-assessed learning success compared to the traditional concept. Furthermore, we wanted to examine the acceptance for the station-based concept in students and residents. 

## Methods

### General Context

The neurology clerkship at the Department of Neurology at the University Medical Center Freiburg usually takes place during the students’ third or fourth year. The mandatory three-week block course includes disease-oriented lectures, symptom-oriented seminars, practical teaching of the neurological examination and bedside teaching. The course ends with a summative multiple choice-question examination for all participants covering all course sections, usually containing two out of 40 questions referring to the NE or its neuroanatomical background. Since the questions of the multiple-choice examinations are made public we could not use the same questions in the next semester making a direct comparison between the results of the students participating in the traditional and the station concepts impossible. At the time of this study no practical assessment for the NE was conducted.

### Design of the Study

The traditional concept was compared to the station concept using a prospective quasi-experimental design using the entire student populations of two consecutive semesters. Primary endpoints of the study were the differences in students’ self-assessments of learning success in six subdomains of the NE, and secondary analyses focused on evaluation results of students and residents. The study was approved by the Ethics Committee of the University Medical Center Freiburg, Germany.

### The traditional concept

One resident taught a small group of 6 (5 to 7) students on two consecutive afternoons for 3 hours each. The content was based on a 24-page script developed for the students and structured in the chapters mental status, cranial nerves, motor system, reflexes, sensory system, and coordination with gait in accordance with published consensus-statements [13], [14]. Residents received an additional 5-page handout listing the 59 single tests they should teach in a structure according to the chapters in the students’ script with short accompanying examples how to perform these tests.

### The station concept

The same content of the NE as in the traditional concept was divided into 8 stations. The examination of the cranial nerves was split in three 25-minute lasting stations “Impaired Seeing” (cranial nerves II, III, IV and VI), “Face” (cranial nerves V and VII) and “Tongue and Throat” (cranial nerves IX, X and XII with extra space for cranial nerves I and XI). Tests for cranial nerve VIII were subsumed under “Coordination”. The station “Motor System” lasted 75 minutes and included tests for muscle tone, strength and reflexes. Tests for the sensory system, coordination and gait were combined into a 40-minute lasting station “Sensation and Coordination”. The stations “Mental Status” (focusing on symptoms concerning alertness, perception, language, concentration and memory) and “Neurologic Examination in Patients with Altered Level of Consciousness” lasted 40 minutes. The closing station “Screening NE” recapitulated the essential steps of the NE [7], [13] and was used to demonstrate and exercise the NE in one sequence. The independence of the content of all stations (except for the closing station “Screening NE”) was an important requirement for the development of the station concept and was carefully paid attention to. For the residents, we developed a specific one-page guideline for every station to provide a structuring and didactical aid. The basic didactic scheme was identical for all stations: After a short introduction by the resident (optionally using the introducing clinical case of the new script, see below) defining the station’s content, its learning objectives, the clinical context and its neuroanatomical background, the resident should demonstrate the tests belonging to the station. Subsequently the students should practice these tests with their peers with immediate feedback from the resident. Afterwards students should be invited to ask remaining questions. Finally, the resident gives a summary of the station with emphasis on the achieved learning objective.

In the station-concept one resident was assigned to teach the content of one station per day resulting in two to six repeats depending on the length of the station. The students’ groups of 6 (5 to 7) students had to rotate through the stations following a default plan (see Table 1 (Tab. 1)): At day one students were instructed alternately in the three cranial nerves stations and the “Motor Station”, at day two the three stations “Sensation and Coordination”, “Neurologic Examination in Patients with Altered Level of Consciousness” and “Mental Status” were instructed in parallel. For the closing station “Screening NE” students remained at the residents who instructed the prior station.

The students’ script of the station concept was identical with regard to content to the script of the traditional concept. The structure was adapted to the stations by assigning one chapter per station. Two new chapters “Neurologic Examination in Patients with Altered Level of Consciousness” and “Screening NE” were introduced by regrouping the existing examination tests. Two short clinical vignettes establishing the clinical context and the learning objectives introduced all chapters. The newly formatted script maintained the length of 24 pages.

### Self-assessment and evaluation of students

Two days after accomplishing the NE-course students of both semesters were asked to voluntarily self-assess their personal learning success for each of the six commonly accepted domains of the NE (mental status, cranial nerves, motor system, reflexes, coordination and sensory system [13], [14]) on a six-level grading scale allowing a comparison between the two concepts. They were also invited to evaluate for motivation, engagement and quality of mentoring of the residents, the group size and the usefulness of the student script on a 6-point-Likert-scale. Furthermore they were asked to evaluate their subjectively achieved competence to perform the basic steps of the NE and the degree of preparation for bedside use of these acquired practical skills. Finally students were asked for an overall rating of the NE course.

### Evaluation of residents

We determined the duration of residency and educational qualifications (attendance of workshops, didactic certificates etc.) for every resident at each time point of teaching the NE. After ending the neurology clerkship we conducted a voluntary online-survey evaluation for each of the teaching concepts: Residents should grade the respective concept of teaching NE that they absolved. We also asked those who participated in both concepts for a comparison of the two concepts.

### Statistical analysis

The Likert-scaled results of the evaluations and self-assessments were statistically analyzed using SPSS 21 (IBM, USA). Data are presented as mean with standard deviation. The p-values were derived from t-tests (significance level 5%, two-tailed). Adjustment for multiple testing was carried out calculating the false discovery rate (FDR).

## Results

Characteristics of participating students and residents are shown in Table 2 (Tab. 2).

144 of 156 students (92,3%) completing the NE course with the traditional concept and 139 out of 153 students (90,8%) completing the NE course with the station concept participated in self-assessments and evaluation. The results of the self-assessed learning success and evaluation results of each cohort are shown in Table 3 (Tab. 3). Eight items were significantly rated better in the station concept: Three items of the self-assessed learning success (“motor system”, “coordination” and “mental status”) and five items of the evaluation (motivation and engagement of the residents, mentoring of the residents, usefulness of the script, feeling of preparedness for clinical practice and the overall-grade).

Ten residents (41,7%) participated in the online-evaluation of the traditional concept. Two residents rated the concept “1= excellent”, seven “2= good” and one “3= satisfactory”, resulting in an average grade of 1,9. In the online-evaluation of the station concept 21 residents (87,5%) participated. Nine rated “1= excellent”, eleven “2= good”, and one “3= satisfactory”, resulting in an average grade of 1,6 with no significant difference to the average grade of the traditional concept. Ten out of 20 residents participating in both concepts rated the station concept better (50%) and eight rated it equally (40%) to the traditional concept. Two residents favored the traditional concept (10%).

## Discussion

Teaching the NE by residents is an often-used approach, because they use the NE in daily routine and it is thought to be a well-defined content making teaching manageable for residents in their first years. In our clinic more than 40% of the residents teaching the NE to undergraduates had less than 2 years of residency and less than 20% of all teaching residents were formally taught in educational skills. On the other hand those who had completed didactical training had more than 3 years of residency. This leads to a distinct inhomogeneity of teaching residents: on one side clinically less experienced residents without educational qualification, on the other side residents with more clinical experience and higher chance of educational qualification. This might especially have an impact on teaching the NE, since it becomes apparent, that it includes an extensive content of tests and corresponding neuroanatomical background knowledge resulting in textbooks devoted exclusively to the NE [15], [16], [17], [18]. In our course traditionally 59 tests were taught, leading to a great amount of teachable content and relevant neuroanatomical background. Teaching this mix of practical skills and complex background knowledge is difficult for clinically less experienced residents, who are not didactically trained. The division of the entire content into stations enabled the residents a focused preparation of specific domains of the NE, resulting not only in a substantiated knowledge in this domain but also in better confidence in the own examination skills. This was shown before to be an important factor for successfully teaching practical skills [19], which likely contributed to the better-evaluated motivation and engagement and the appreciated feeling of good mentoring. Being better prepared in contents also allowed residents to concentrate more on didactic aspects of teaching. Since only a minority of residents completed an educational training we provided a basic didactic structure that was individualized for the content of each station and served as a guideline for teaching. Interestingly, due to the repetitions of the stations, residents noticed a learning curve in their didactical skills (personal communication). This is reflected by the preference for the station concept by 50% of the residents, while only 10% favored the traditional concept.

As primary outcome for students’ learning success we used aggregated self-assessments, which were recently demonstrated to have a good correlation with external standards for estimation to the effectiveness of new teaching programs [20], [21]. The aggregated self-assessments in our study showed a significant increase in three of the six domains of the NE taught with the station concept. Teaching the extensive and complex examining steps for motor function (tests for muscle tone and strength) and coordination (including gait) did benefit from the structured approach. The self-assessed learning success of examination of the mental status can be most likely contributed to the above-described better preparation and improvement of teaching techniques of the residents, since especially the examination of the mental status is a rather abstract and challenging topic for teaching. Three domains of the NE (Reflexes, Cranial nerves and Sensation) were already rated close to maximum in the traditional concept, maybe because these domains offer an intrinsic framework for teaching. These domains therefore offered very little room for improvement and there was no significant difference with the station concept.

With regard to the students’ evaluation results there are several notable points. The students evaluated the station concept significantly better in their feeling of being prepared for clinical practice although their feeling of competence to conduct the basic examination steps did not differ between the two groups. We believe that creating a clinical context by introducing the short clinical vignettes of the adapted script as an introduction for each station contributed to this increase, since in clinical practice only a selection of the 59 tests are needed in a patient, depending on the actual complaints. This is already a step towards the hypothesis-driven approach of the NE [9]. Furthermore, structuring the initially overwhelming quantity of neurological tests in smaller subdivision may also contribute to the better overall evaluation results. This conclusion is supported by significantly better evaluation results for usefulness of the new script that with regards to content remained identical to the script of the traditional concept, but in structure was aligned to the station concept.

There are limitations to this study. We did not determine the baseline skills of the participants, but since neurology is taught at only this particular time in our curriculum we assumed that the participants of our study did not differ much in their previous neurological knowledge and experience. Since we used aggregated self-assessment and evaluations of students as main outcome measure we could not demonstrate better practicing of students in neurological tests which is usually assessed with an OSCE, but early work by Anderson et al. [22] demonstrated good correlations for aggregated self-assessments of neurological skills with results assessed with an OSCE. In addition, this study was conducted to provide a fair basis for an OSCE since obvious differences in teaching qualifications did exist in the traditional system. Furthermore, by controlling for group size, which remained constant in both concepts and showed similar results in the evaluation of both concepts, we can demonstrate that the evaluations and self-assessments are reliable methods to differentiate between unchanged and changed results. The highly rated motivation and engagement of the residents may be partially explained by the Hawthrone effect, but the continuing excellent evaluations of the now well-established station concept in our clinic point to a true result.

## Conclusions

The station concept proved to be a successful teaching tool improving the students’ self-assessed learning success and acceptance compared with the traditional concept, especially for those aspects that were regarded as more complex. Furthermore, by reducing the teaching content for residents, providing basic didactic support and enabling repeated teaching of the same content in a short period of time it also offers a good chance especially for clinically and didactically less experienced residents to improve teaching skills. The effort for converting a traditional NE course in a station concept course is relatively low but it creates the requirements for a fair assessment of students in an OSCE. At our institution we successfully proceeded with the station concept and have finalized an OSCE for the NE to start next semester, additionally to the theoretical multiple choice examination to better represent the field of Neurology.

Since the NE is a fundamental and essential step in diagnosing and treating patients with neurological disorders, this new teaching concept may be another effective step for making neurology more attractive to students. 

## Competing interests

The authors declare that they have no competing interests.

## Figures and Tables

**Table 1:**
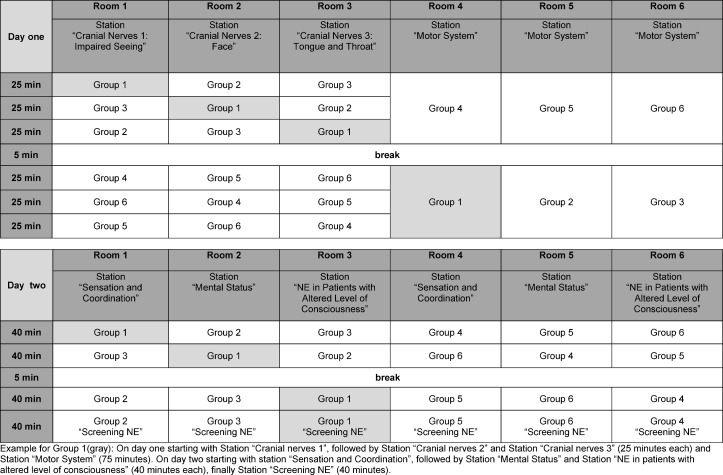
Rotational plan for the station-concept with 6 small groups.

**Table 2:**
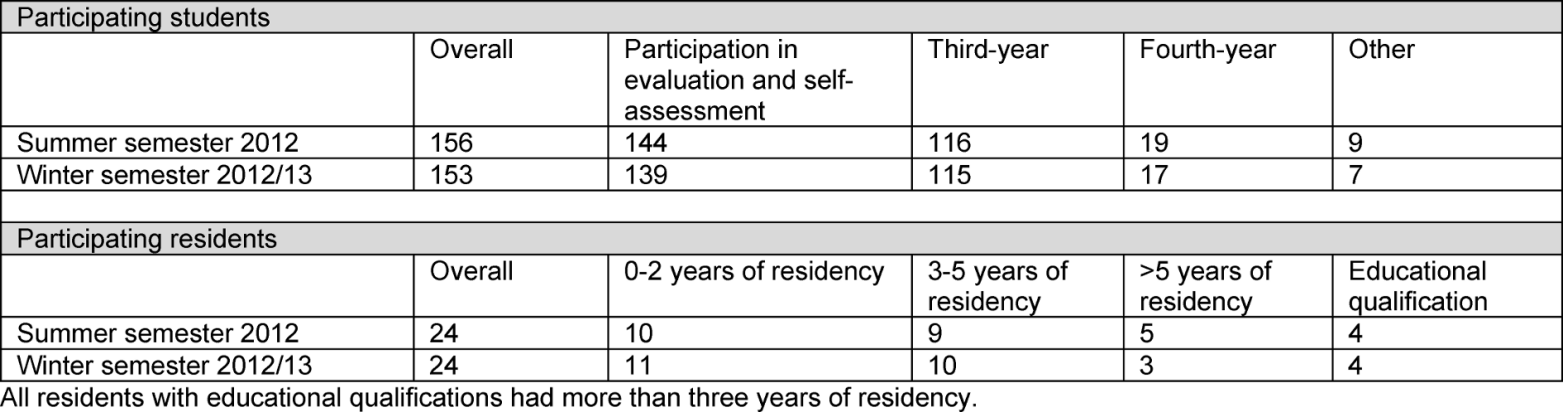
Characteristics of participating students and residents in the two cohorts (summer semester 2012 and winter semester 2012/13).

**Table 3:**
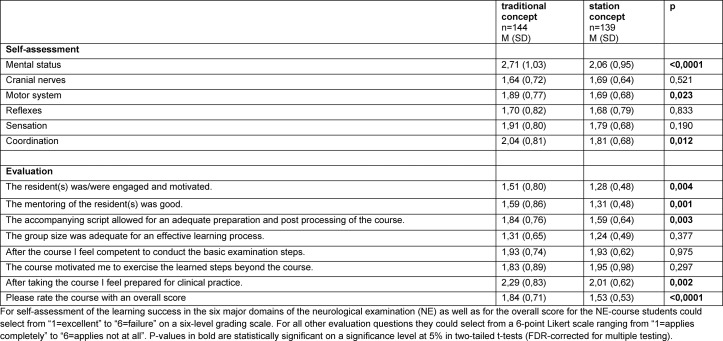
Students’ self-assessment and evaluation results.
